# Effective Control of COVID-19 in South Korea: Cross-Sectional Study of Epidemiological Data

**DOI:** 10.2196/22103

**Published:** 2020-12-10

**Authors:** Gwang Hun Jeong, Hyo Jeong Lee, Jinhee Lee, Jun Young Lee, Keum Hwa Lee, Young Joo Han, Sojung Yoon, Seohyun Ryu, Da Kyung Kim, Myung Bae Park, Jae Won Yang, Maria Effenberger, Michael Eisenhut, Sung Hwi Hong, Andreas Kronbichler, Ramy Abou Ghayda, Jae Il Shin

**Affiliations:** 1 College of Medicine Gyeongsang National University Jinju Republic of Korea; 2 College of Medicine Yonsei University Seoul Republic of Korea; 3 Department of Psychiatry Yonsei University Wonju College of Medicine Wonju Republic of Korea; 4 Department of Nephrology Yonsei University Wonju College of Medicine Wonju Republic of Korea; 5 Department of Pediatrics Yonsei University College of Medicine Seoul Republic of Korea; 6 Department of Pediatrics Samsung Changwon Hospital, Sungkyunkwan University School of Medicine Changwon Republic of Korea; 7 College of Medicine Yonsei University Wonju College of Medicine Wonju Republic of Korea; 8 Department of Gerontology Health and Welfare Pai Chai University Daejeon Republic of Korea; 9 Deparment of Internal Medicine I Gastroenterology, Hepatology, Endocrinology & Metabolism, Medical University of Innsbruck Innsbruck Austria; 10 Luton & Dunstable University Hospital NHS Foundation Trust Luton United Kingdom; 11 Department of Global Health and Population Harvard TH Chan School of Public Health Boston, MA United States; 12 Department of Internal Medicine IV Nephrology and Hypertension, Medical University of Innsbruck Innsbruck Austria; 13 Urology Institute University Hospitals System, Case Western Reserve University School of Medicine Cleveland, OH United States

**Keywords:** COVID-19, Korea, strategies, epidemiological characteristics

## Abstract

**Background:**

South Korea is one of the few countries that has succeeded in flattening the curve of new COVID-19 cases and avoiding a second outbreak by implementing multiple strategies, ranging from an individual level to the population level.

**Objective:**

We aim to discuss the unique strategies and epidemiological characteristics of COVID-19 in South Korea and present a summary of policies implemented by the Korean government during the COVID-19 pandemic.

**Methods:**

We designed a cross-sectional study of epidemiological data published by the Korea Centers for Disease Control and Prevention on October 1, 2020. We analyzed detailed epidemiological information of COVID-19 cases, including the number of confirmed cases and resulting deaths.

**Results:**

As of October 1, 2020, a total of 23,889 confirmed COVID-19 cases and 415 deaths were reported in South Korea. In this paper, we present data on the epidemiological characteristics and transmission of the disease and discuss how the South Korean government, health care providers, and society responded to the COVID-19 outbreak.

**Conclusions:**

Understanding the epidemiological characteristics of COVID-19 in South Korea and the government’s successful efforts in managing the spread of the disease can provide important insights to other countries dealing with the ongoing pandemic.

## Introduction

In December 2019, several cases of pneumonia of unknown origin were reported in Wuhan, China. The causative agent was identified to be a new coronavirus, which was tentatively named as the 2019 novel coronavirus (2019-nCoV) [1]. The disease continued to spread rapidly across China, and on January 30, 2020, the World Health Organization (WHO) announced the outbreak was a public health emergency of international concern [2]. On February 11, 2020, the Coronaviridae Study Group of the International Commission on Taxonomy of Viruses named 2019-nCoV as SARS-CoV-2, and the WHO officially named the disease caused by this virus as COVID-19 [3].

**Figure 1:**
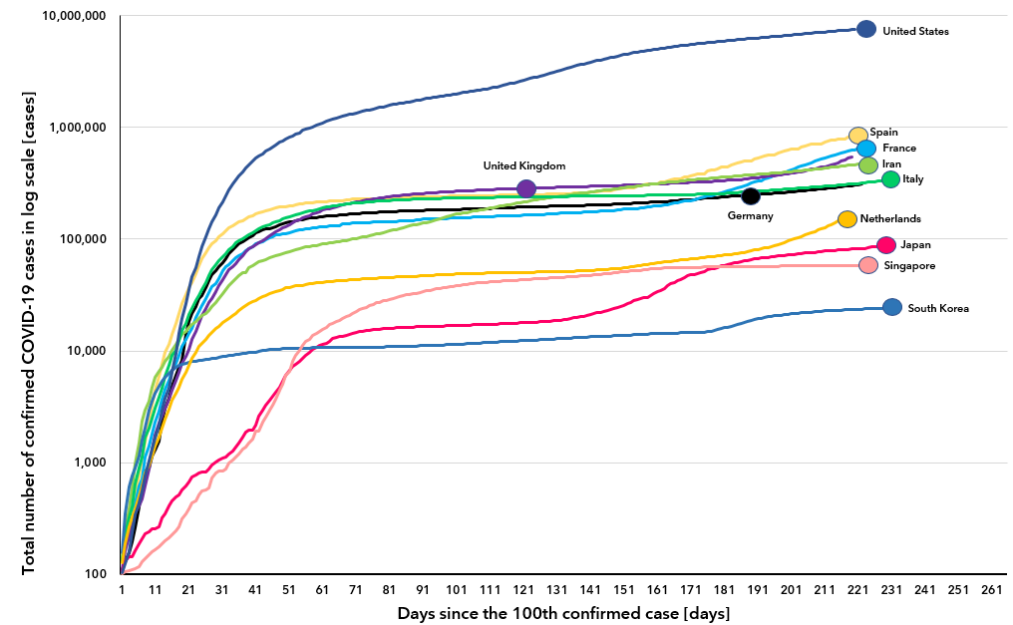
Cumulative number of confirmed COVID-19 cases (log scale) by days since the 100th confirmed case.

**Figure 2:**
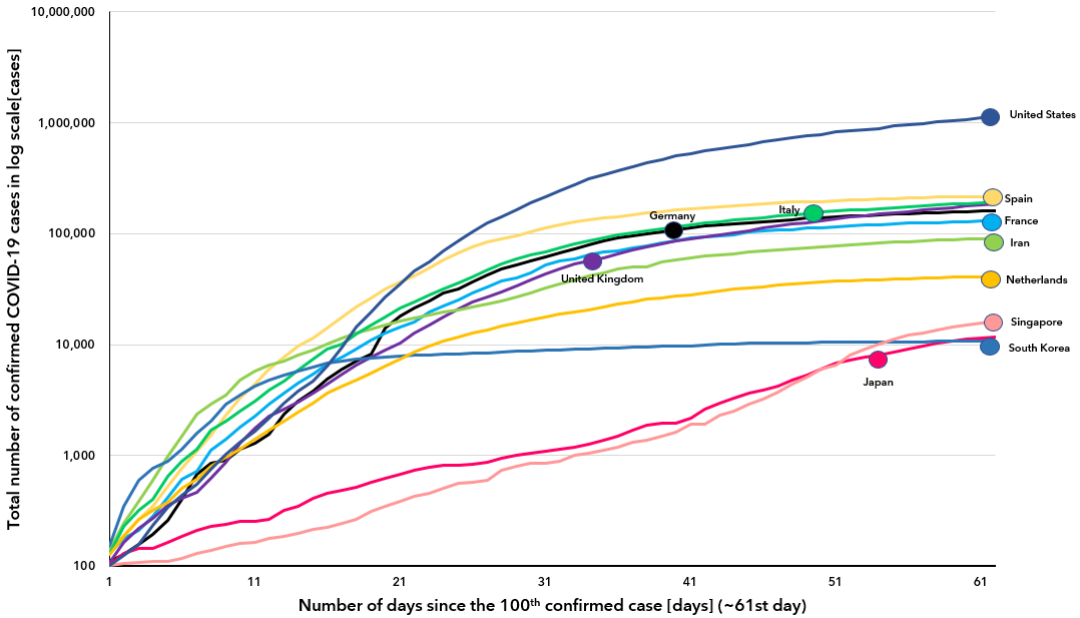
Cumulative number of confirmed COVID-19 cases (log scale) by days since the 100th confirmed case (x-axis cut up to 61 days).

The WHO declared COVID-19 as a pandemic on March 11, 2020. As of October 1, 2020, more than 33,558,134 cases had been confirmed by laboratory tests globally, and more than 1,004,979 COVID-19–related deaths were reported [4]. According to the WHO, the Republic of Korea was the second-most affected country worldwide as of March 9, 2020 [5]. Since then, the daily number of newly confirmed COVID-19 cases have decreased rapidly. As of October 1, 2020, in South Korea, a total of 23,889 COVID-19 cases were diagnosed, with the country ranking 50th in terms of the total number of confirmed cases worldwide [4]. Although a second peak was observed around August 15, South Korea is one of the few countries in the world to have successfully maintained a flat infection curve for more than 50 days (Figures 1 and 2). Given the rapid spread of COVID-19, other countries are likely to experience similar outbreaks like South Korea. Thus, it is noteworthy to retrospectively review how the COVID-19 outbreak spread in South Korea and how the country managed to eventually flatten the curve of the number of COVID-19 cases. In this study, we present the current status of COVID-19 in South Korea, including data on the transmission of the disease, its epidemiologic characteristics, and the government and society’s responses toward the outbreak. We believe the findings of this study would help other countries in their efforts to control and stop the outbreak of COVID-19. To our knowledge, our study is the first to report the epidemiological characteristics of COVID-19 transmission and the government’s policies for disease control.

## Methods

We collected demographic and epidemiologic data from COVID-19 reporting and surveillance data published by the WHO and Korea Disease Control and Prevention Agency (KDCA), formerly Korea Centers for Disease Control and Prevention (KCDC), from January 20, 2020, to October 1, 2020. The official COVID-19 reports in Korea were updated twice a day until March 1, 2020, by the Central Disaster and Safety Countermeasures Headquarters (CDSCHQ) of KDCA. Thereafter, the reports were updated only once a day. The English version of these reports are also available from the KDCA website’s English home page, which are updated daily by the Division of Risk Assessment and International Cooperation of KDCA [6].

In addition, we reviewed various government policies and responses to COVID-19 based on these daily updates, up to October 1, 2020. Where additional information was needed, we reviewed and confirmed the data released by the press.

## Results

### Spread of COVID-19 Cases in South Korea

**Figure 3:**
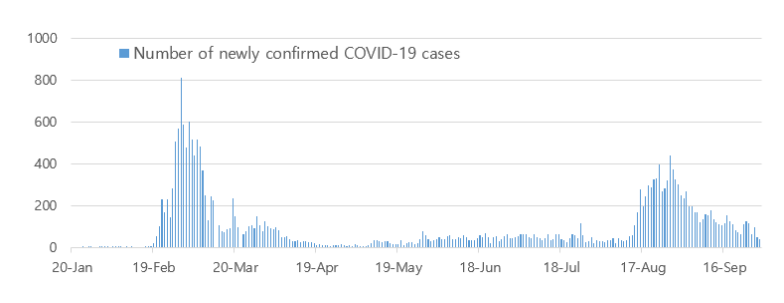
Number of newly confirmed COVID-19 cases per day in South Korea.

The first confirmed COVID-19 case in South Korea was reported on January 20, 2020 [7]. After the first outbreak among a religious group was reported on February 19, the number of COVID-19 cases rapidly began to spread and resulted in mass infection in the country. The highest number of confirmed cases per day was 813, as recorded on February 29, 2020 [4]. However, as of March 15, 2020, the number of confirmed COVID-19 cases per day has dropped by about 76 (Figure 3). South Korea was able to take control of the spread of COVID-19 without enforcing a lockdown and managed to flatten the curve and successfully maintain it like so until August 13 (Figures 1 and 2). Because of a massive antigovernment rally and the relaxation of the deterrence policy, a second outbreak emerged, with the number of confirmed COVID-19 cases per day increasing to 441 as of August 28, 2020. Finally, the number of confirmed COVID-19 cases per day substantially dropped again, with about 113 cases reported as of September 30, 2020.

**Figure 4:**
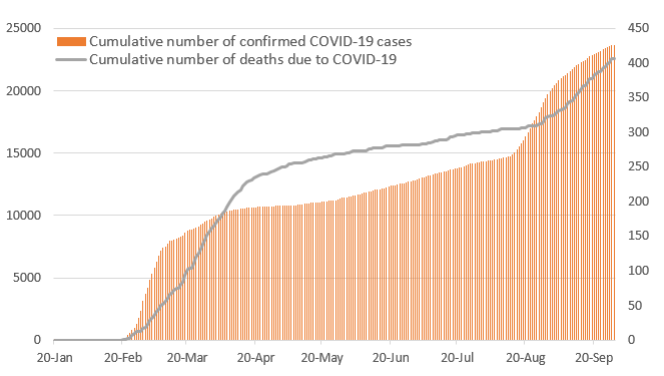
Cumulative number of confirmed COVID-19 cases and resulting deaths in South Korea.

**Figure 5:**
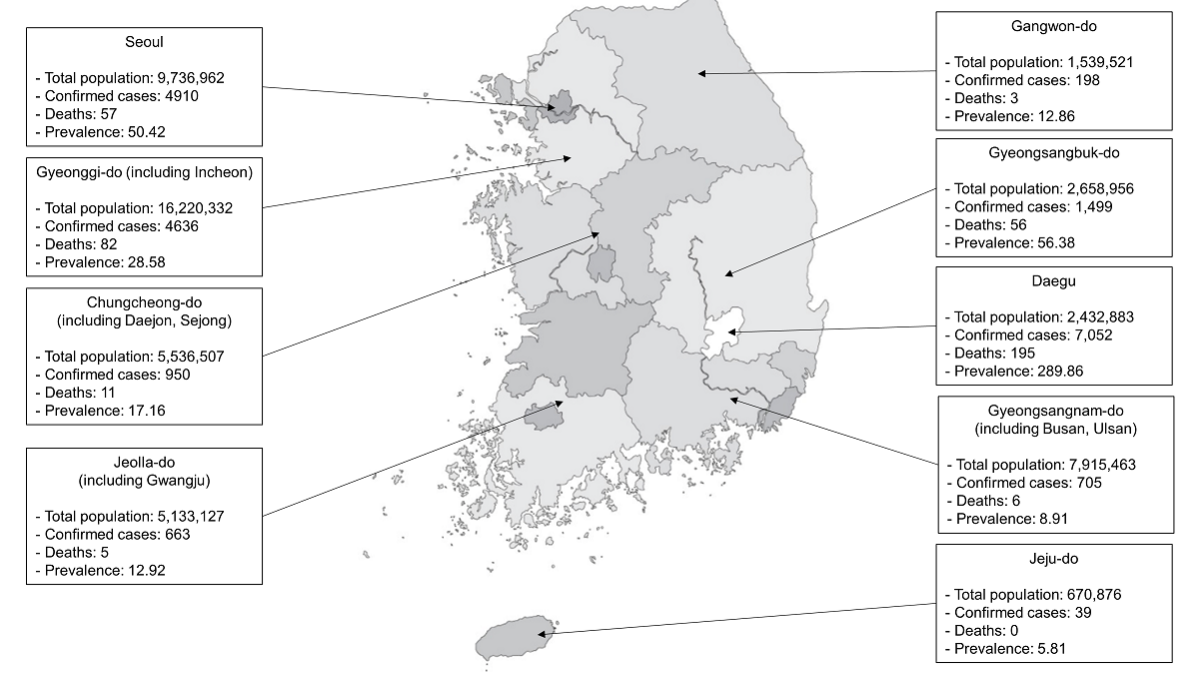
Prevalence of COVID-19 across various regions in South Korea (midnight, October 1, 2020).

As of midnight, October 1, 2020, a total of 2,328,435 patients had been tested, of which 23,889 (1.03%) patients were confirmed to have COVID-19 [4,8]. Among these patients, 21,666 (90.7%) were successfully treated and discharged from the hospital, 1,808 (7.56%) were still being isolated [8], and 415 (1.7%) died (Table 1). The detailed epidemiological characteristics of patients with COVID-19 in South Korea are presented in Table 1. Of the 415 total deaths, 195 (47%) were reported in Daegu (Figure 4). The highest prevalence rate for COVID-19 (289.86) was reported in Daegu. In contrast, a relatively low prevalence of 50.42 was reported in Seoul, which has the largest population as a single city (Figure 5).

**Table 1:** Detailed epidemiologic characteristics of patients with confirmed COVID-19 in South Korea as of midnight, October 1, 2020 (N=23,889).

Characteristic			Value, n (%)
**Gender**			
	Male		11,007 (46.1)
	Female		12,882 (53.9)
**Age range, years**			
	0-9		584 (2.4)
	10-19		1310 (5.5)
	20-29		4769 (20.0)
	30-39		2917 (12.2)
	40-49		3192 (13.4)
	50-59		4434 (18.6)
	60-69		3795 (15.9)
	70-79		1913 (8.0)
	80 and above		975 (4.1)
**City**			
	Seoul		5323 (22.3)
	Busan		427 (1.8)
	Daegu		7133 (30.0)
	Incheon		919 (3.9)
	Gwangju		495 (2.1)
	Daejeon		360 (1.5)
	Ulsan		147 (0.6)
	Sejong		76 (0.3)
	Gyeonggi-do		4405 (18.4)
	Gangwon-do		224 (0.9)
	Chungcheongbuk-do		172 (0.72)
	Chungcheongnam-do		487 (2.0)
	Jeollabuk-do		128 (0.5)
	Jeollanam-do		170 (0.7)
	Gyeongsangbuk-do		1566 (6.5)
	Gyeongsangnam-do		291 (1.2)
	Jeju-do		59 (0.3)
Quarantine system			1517 (6.4)
**COVID-19 cases from overseas**			
	China		23 (0.1)
	Asian (except China)		1596 (6.7)
	Europe		574 (2.4)
	Africa		945 (4.0)
	America		84 (0.5)
	Oceania		15 (0.1)
**Deaths due to COVID-19**			
	Total deaths		415 (100.0)
	**Gender**		
		Male	219 (52.8)
		Female	196 (47.2)
	**Age range, years**		
		30-39	2 (0.5)
		40-49	4 (1.0)
		50-59	19 (4.6)
		60-69	44 (10.6)
		70-79	138 (33.3)
		80 and above	208 (50.1)

KCDC is a public health institution that plays an important role as the headquarters of the Centers for Disease Control. This organization aims to investigate the occurrence patterns and epidemiology of COVID-19, care for patients with a confirmed diagnosis, and individuals they have been in contact with and accordingly inform people of appropriate measures to prevent the spread of disease. Since it raised the national infectious disease crisis level for COVID-19 to “serious” on February 23, 2020, the Korean government has established the CDSCHQ, operated by the Ministry of Health and Welfare and Ministry of Public Administration and Security, to support KCDC with its disease control efforts and to provide the necessary assistance in matters requiring coordination between the central government and local municipal governments.

Along with KCDC and CDSCHQ, the Korean government has responded to changes in situations every single day and put forward various policies to prevent the spread of COVID-19 in the society (see Table S2 in Multimedia Appendix 1).

### Policies and Responses of the South Korean Government

#### Measures to Prevent Entry of the Virus

Among the many countries in the world where COVID-19 outbreaks have occurred, Korean policies concerning immigration restrictions and bans are the least restrictive. Currently, South Korea enforces immigration bans only for passengers who possess Chinese passports issued by Hubei Province, all foreigners who have visited Hubei within the past 14 days, and passengers of the anchored cruise ship (Diamond Princess) at Yokohama Port in Japan. Moreover, guidelines for passengers arriving from China have been changed to mandate a visa for transfers via Korea. The visa-exemption entry of Japanese passport holders was restricted, and the validity of all previously issued visas was suspended. For all passengers arriving in Korea from different parts of the world, entry is allowed under conditions that these foreign nationals submit a special in-flight quarantine health status report and install a mobile app for COVID-19 self-diagnosis that provides a daily report of their health status for 14 days. Since March 22 and 25, 2020, the Korean government has been conducting COVID-19 diagnostic tests for every inbound traveler entering the country from Europe and the United states, respectively. On the first day of conducting these diagnostic tests, 1444 individuals underwent the tests and 19 of them were confirmed to have COVID-19 [8].

#### Early Detection Through Screening Clinics

The Korean government’s policy against COVID-19 was to invest as many medical resources as possible for screening and early diagnosis of disease [9]. This is important because isolating and monitoring patients with confirmed COVID-19 in early stages of the disease can impede further disease transmission and enable patients to be transferred to the hospital soon after they show severe respiratory symptoms, thereby reducing mortality and morbidity.

On February 7, 2020, the Korean government approved test kits that could produce results in 6 hours; thereafter, supply of the diagnostic test kits increased from 200 to 3000, and the number of screening clinics increased from 288 to 556 [10]. By February 26, South Korea had tested 46,127 individuals, while at that timepoint, Japan had tested only 1846 individuals and the United States has tested only 426 individuals [11]. South Korea continued to perform a higher number of screening tests than most other countries, with 2,322,999 tests conducted until October 1, 2020 [6].

To prevent hospitals from being overwhelmed, the Korean government opened more than 600 testing centers designed to screen as many people as quickly as possible and to keep health workers safe by minimizing contact. The government also introduced drive-through screening clinics in February. At that time, these clinics were operational in more than 50 locations nationwide, which helped minimize contact between medical staff and individuals undergoing tests and thus reduce cross-contamination and test administration time (from almost 1 hour to only about 10 minutes). These drive-through testing clinics allowed more than 15,000 COVID-19 laboratory tests to be conducted per day [9]. At some walk-in centers, patients enter a chamber resembling a transparent phone booth, wherein health care workers administer throat swabs using thick rubber gloves built into the chamber’s walls.

#### Systematic Diagnostic Testing

COVID-19 suspected cases are confirmed only by laboratory tests, using any of the following pathogenic detection criteria: (1) a positive test for SARS-CoV-2 based on respiratory or serum specimen examined by real-time reverse transcription polymerase chain reaction or (2) full genome sequences of the virus isolated from the respiratory or serum specimen are identical to SARS-CoV-2 genome sequences [12]. Patients who undergo laboratory diagnostic tests are classified into 2 groups: The first group “patients with suspected COVID-19” comprises those who have been in contact with a patient with confirmed COVID-19 within the last 14 days and have symptoms of fever over 37.5℃ or respiratory symptoms (including cough, dyspnea, and acute respiratory distress syndrome); these patients are prioritized among those who are eligible for laboratory examination [12]. The second group “patients under investigation” comprises those who have visited countries with a high incidence of COVID-19, such as China, Hong Kong, and Macao within the last 14 days or those who are epidemiologically related to the domestic outbreak and show COVID-19–related symptoms; these patients take priority after the suspected patients [12]. Those who have unexplainable pneumonia and are suspected to have COVID-19 according to the doctor’s opinion are also classified as patients under investigation and can receive diagnostic tests, even if they do not have a history of exposure to patients with confirmed COVID-19 [12]. In addition, individuals who have been in contact with patients with COVID-19 may be tested for the purpose of releasing them from quarantine even if they have no symptoms, and some of them may be identified as asymptomatic patients [12].

In the beginning, the diagnostic criteria were so strict that only a few people could receive diagnostic tests, which resulted in many patients having diseases with an unclear diagnosis. Some COVID-19 cases were confirmed without a history of exposure to patients with confirmed infection or travel within high-risk countries, or expression of any clinical symptoms; therefore, on February 16, 2020, the government decided to expand the range of patients that could receive a diagnostic examination considering the high risk of human-to-human COVID-19 transmission due to asymptomatic patients [6].

#### Contact Monitoring and Epidemiological Investigations Using Information Technology

COVID-19 contact investigation and management involved the following aspects: (1) determination of the location of the contact (patient route), (2) exposure risk evaluation, (3) contact classification, and (4) contact management [9]. Exposed individuals (hereafter, contacts) need continuous monitoring regardless of whether they exhibit symptoms. The range of contact is determined by the local city and provincial response teams who evaluate the symptoms of patients with confirmed infection, whether or not they were wearing a mask, their exposure situation, and other factors. In general, the range of contact is set to 24 hours before the patient with confirmed infection begins to exhibit symptoms. The Korean government is undertaking vigorous measures to track and test those who had been in contact with these patients, by conducting only a proxy interview with the patient.

Objective methods are also used for contact verification, including use of a GPS and review of medical facility records, card transaction logs, and closed-circuit television recordings. Since the government tracks the movements of patients with confirmed COVID-19 in detail and discloses them to the public via a smartphone app, users can determine whether or not they have been in contact with the patient themselves [13]. If these contact individuals do not have any notable symptoms, he or she is released from the mandatory quarantine period a day after a fortnight has passed from the date of final contact with the patient with confirmed diagnosis. In cases where the patient was residing with a family, de-isolation was permissible 14 days after the patient was de-isolated. Individuals violating self-isolation guidelines were imposed with a fine of up to US $1000 or imprisonment for up to 1 year [9].

#### Quarantine, Isolation, and Surveillance

If the health center recognizes a patient with confirmed COVID-19 first, it reports to the provincial government and KCDC immediately. Thereafter the health center evaluates the severity of the case by checking the patient’s level of consciousness, body temperature, respiration rate, and other high-risk indicators to decide which of the following isolation methods would be most appropriate: (1) self-isolation, (2) facility isolation, or (3) hospital isolation [9]. If the patient has no severe symptoms, no problem with arranging for daily essential supplies, and can live independently, he or she is eligible for self-isolation. However, if the patient has difficulties in living independently, does not have a proper residence, or lives with relatives belonging to a high-risk group (ie, people aged over 65 years, having underlying diseases, or requiring oxygen treatment due to reduced oxygen saturation <90% in indoor settings), he or she should be admitted to a life care center. If the physician in charge determines that the patient’s disease severity is acceptable for his or her transfer to a life care center, a hospitalized patient can be moved into facility isolation. Patients belonging to the high-risk group or having high fever (>38.5℃) require hospital isolation [9].

#### Accessible and Effective Medical Intervention

South Korea has a National Health Insurance System (NHIS) that covers more than 50 million people, that is, over 97.1% of the entire nation’s population, whereas the remaining 2.9% are supported by the Medical Aid Program [14]. The NHIS covers most cases of diagnosis and treatment related to COVID-19, and these insured patients can easily access health facilities with considerably lower medical expenses than those incurred by insured patients in other countries. Patients with COVID-19 are supported by the NHIS, central government, and local governments. Of the total medical expenses, the government and local governments support the expenses incurred by the patients, after subtracting NHIS’ levy of the total medical expenses, as determined by Korea’s health insurance system.

At first, there were no approved antiviral drugs for COVID-19 treatment in Korea, but later, the government approved some antiviral agents such as remdesivir, an anti-Ebola drug, that were likely used for COVID-19 treatment in other countries,. Patients with confirmed COVID-19 are treated with symptomatic therapy comprising fluid replacement, antipyretics, oxygen therapy, or antibiotics to prevent secondary infections. Individuals who have been in contact with these patients do not require any special treatment until they show COVID-19–related symptoms [15].

#### Realignment of Health Care Institution Use System

To reinforce infection control within health care institutions, the Korean government has been providing infection control guidelines to these institutions and focusing on expanding cooperation and communication within the health care circles. Health care institutions are required to provide patients with guidance on hygiene, restrict the entry of visitors and control visiting conditions, screen each visitor’s body temperature, and mandate the wearing of masks [16].

The Korean government designated 60 institutions for hospitalized treatment of patients with COVID-19, managed the supply empirical therapies, and shared distributor information. Moreover, the Korean government is trying to ensure non–COVID-19 patients’ safety and accessibility to hospitals by separating areas for patients with respiratory illnesses from those for patients with other illnesses. As of September 30, 270 hospitals in Korea were designated as COVID-19 protection hospitals, as a result of which many non–COVID-19 patients were not inconvenienced in using medical institutions [10]. The South Korean government also allows physicians to offer over-the-phone medical consultation and prescriptions temporarily, until the COVID-19 outbreak ends. Telemedicine services were previously considered illegal in South Korea, but the government effected a decision to allow telemedicine in order to lower the risk of COVID-19 infection by reducing contact between patients and health care workers.

#### Prompt Amendments of Associated Laws

According to a previous study [1] on the Infectious Disease Prevention and Management Act, the Minister of Health and Welfare or the head of a local government may restrict the assembly, in order to prevent the spread of infectious diseases and may impose a fine up to ₩ 3 million (US $2382.27 as of March 18, 2020) to those in violation of the prohibited measures. During the COVID-19 outbreak, the local governments identified some groups that held large-scale rallies and penalized them. In addition, the Ministry of Health and Welfare reorganized the entire quarantine system to effectively respond to the COVID-19 outbreak and supplemented the necessary measures to respond to an infectious disease outbreak. Thus, 3 amendments to the Act on the Prevention and Management of Infectious Diseases, the Quarantine Law, and the Medical Law were urgently proposed, and these became effective after they were passed in the plenary session of the National Assembly on February 26, 2020 [17]. The current tools and policies are based on the “Infectious Disease Control and Prevention Act,” the legal framework for the Korea’s disease-prevention policy which was revised in 2015 after the outbreak of Middle East respiratory syndrome. South Korea’s experience with this infectious disease enabled rapid amendments of associated laws. The detailed points of the amendments to the law are presented in Table S2 and supplementary text in Multimedia Appendix 1.

#### Public-Private Co-operation and Civic Awareness

COVID-19 is known to be spread through droplets generated by cough or sneezing and by touching one’s eyes, nose, or mouth with contaminated hands. In addition, aerosols and feces are known to be associated with the spread of the disease [18-20]. Considering these facts, the Korean government has suggested various methods for preventing the spread of COVID-19; these include personal hygiene management, the use of masks, and practicing social distancing. In particular, due to the shortage of masks for the public, the government made a policy to enable people to purchase 2 masks per week per person from March 9, 2020. The detailed policies undertaken by the government to enhancing personal hygiene measures are presented in the Table S2 and supplementary text in Multimedia Appendix 1.

However, even if the public adheres to all the above-mentioned preventive measures, doctors who consult outpatients are still at high-risk of exposure. In order to reduce their risk, the government has installed temperature-screening devices at the entrances of hospitals and asked all visitors to complete a self-questionnaire to be able to record their contact history and travelling history, as well as fever and respiratory symptoms. In addition, outpatient doctors are required to wear personal protective equipment, including a mask, and to maintain a distance of at least 2 meters from the patient [16]. For instance, Severance Children’s Hospital in Seoul was able to successfully adopt this policy to contain the spread of infection from the outpatient to inpatient departments (see supplementary text in Multimedia Appendix 1).

## Discussion

In this study, we aimed to discuss how COVID-19 spread in the community and health care institutions across South Korea; review epidemiological characteristics of the disease; and discuss how the government, health care providers, and society responded to effectively prevent the spread of the disease. Although SARS-CoV-2 has rapidly spread across the word, affecting countries such as the USA, Italy, Spain, and the UK, South Korea is the only country that has successfully flattened the curve and managed to maintain a plateau with regard to new COVID-19 infections even despite large outbreaks. Moreover, it has managed to do so without China’s draconian restrictions on speech and movement, or lockdowns with economic consequences such as those in Europe and the USA [13,21].

The South Korean government has been known for its effective response to COVID-19, and the general public has been strictly following quarantine guidelines [22]. Since the start of the COVID-19 outbreak in Korea, health care workers as well as the general public have been using masks and hand sanitizers and practicing social distancing. As a result of these efforts, the number of human contacts that a single resident makes per day has been reduced to almost a hundredth compared to that before the outbreak. In addition, thorough its quarantine and contact tracing system, the government makes an effort to identify undiagnosed patients immediately after patients receive a confirmed diagnosis, by tracking their route and seeking the infection source. Once someone is identified as a contact, he or she is immediately asked to undergo self-isolation and their health status is continuously monitored by the government. Thus, they can be diagnosed in a timely manner and receive treatments as soon as they show any relevant symptoms, thereby lowering mortality and morbidity. Furthermore, through a system such as a drive-through screening clinic, a large number of diagnostic tests can be conducted quickly without threatening the safety of patients and doctors. With these systems in place, even asymptomatic patients are not missed, thus making Korea’s COVID-19 statistics more reliable. With a population of 50 million, the country has substantially slowed down the spread of the COVID-19 epidemic. South Korea reported only 113 new COVID-19 cases as of October 1, 2020, down from 813 cases at its first peak on February 29, 2020, and 441 at its second peak on August 27, 2020 [4]. In particular, Seoul, which is the most densely populated city in South Korea, effectively prevented COVID-19 transmission, with only 5,323 (23%) confirmed cases reported among a population of more than 9 million.

On April 11, 2020, the South Korean government announced a new decision to use electronic wristbands for residents who violate self-isolation rules, such as going outside without notice and not answering phone calls. Although South Korea seems to have succeeded in flattening the curve of COVID-19, some concerns regarding privacy and human rights continue to persist [23]. The Korean public still broadly supports the government’s aggressive contact tracing and strict quarantine guidelines because they believe these measures help prevent transmission and maintain a safe public health system. Amid the prolonged COVID-19 crisis, these measures should be accompanied with a transparent and mature consensus process by the members of society in order to protect the community and its people.

## Appendix

Multimedia Appendix 1 Epidemiological data for the study.

## Abbreviations

CDSCHQ: Central Disaster and Safety Countermeasures Headquarters
KCDC: Korea Centers for Disease Control and Prevention
KDCA: Korea Disease Control and Prevention Agency
NHIS: National Health Insurance System
WHO: World Health Organization
