# Supplementary material for: Effective Control of COVID-19 in South Korea: Cross-Sectional Study of Epidemiological Data
Source: J Med Internet Res. 2020 Dec 10;22(12):e22103. doi: 10.2196/22103 (PMC7732355; doi:10.2196/22103)
Supplement: Multimedia Appendix 1 [file jmir_v22i12e22103_app1.pdf]

Supplementary Material for

Effective Control of COVID-19 in South Korea: Cross-Sectional Study of Epidemiological Data

This file includes:

Supplementary Text
Tables S1, S2

Supplementary Text

Prompt amendments of associated law

The main points of the changed law are as follows.

Act on prevention and management of infectious diseases:

- In case of violation of hospitalization or quarantine measures, the punishment was strengthened to imprisonment for not more than one year or a fine not exceeding 10 million won (7,943.63 USD, 18th March 2020).

- A provision was made to allow the Minister of Health and Welfare to prohibit the export of quasi-drugs, medicines, etc. when a first-class infectious disease is prevalent.

- The manpower of the central government's epidemiological investigators has expanded significantly from 30 to more than 100, and the head of the city's county has been given authority to appoint epidemiological inspectors and quarantine officers.

Quarantine law:

-The quarantine investigation target is subdivided into aircraft, ships, and land, and the quarantine information system is linked to the systems of related organizations that have immigration information and passport information.

Medical law:

-Create an infection monitoring system for patients, guardians, and medical institution workers in medical institutions to strengthen the response system by creating an infection monitoring system.

Public-private cooperation and civic awareness

When coughing, covering mouth and nose with tissues or clothing sleeves is needed. Unlike other countries like the United States and Germany, Korea recommends the general public to wear a mask of any kind. It is because wearing a mask can prevent other people from getting infected when he or she is infected with COVID-19 and have respiratory symptoms. The government has a policy to enable people to purchase two public masks per week per person from March 9, and various smartphone applications have been developed to inform people about mask-selling places such as pharmacies and post offices and the number of masks remaining in each retailer in real time.

As a prevention method, the concept of "keeping social distance" was also newly introduced. This is a strategy to control the spread of infectious diseases by refraining from attending various events in closed spaces and canceling public and private gatherings. Assuming that the amount of social contact has been reduced by one-hundredth compared to before the occurrence of COVID-19, it is predicted that the increase in the disease will stop at 9,400 patients in Korea on April 23rd. The predictive graph is most similar with the current outbreak trend.

It was instructed for outpatient doctors to be equipped with protective equipment including a mask and to be placed at least two meters from the patient. As a successful example of this policy, it can be mentioned what happened at Severance Children's Hospital in Seoul. A father visited the hospital's outpatient clinic with his child on February 29th and later, he was

diagnosed with COVID-19 on March 5th. As February 29th was the day before symptoms occurred, all those who contacted him on that day had to be classified as contactors and be isolated, and even the whole hospital could have been closed. However, the number of contactors in Severance Hospital became zero because all medical workers wore masks and protective equipment and did strict hand hygiene with keeping a distance of over two meters from the patients.

Table S1. Summary of the first case reported in each region

Region	Date	Sex	Age	Origin
Seoul	1/21/2020	M	55	Travel history of Wuhan, China
Busan	2/21/2020	M	19	Oncheon Church
Daegu	2/18/2020	F	61	Shincheonji Church
Incheon	1/20/2020	F	35	Chinese, Living in Wuhan, China
Gwangju	2/4/2020	F	42	Unknown, travel history of Thailand
Daejon	2/21/2020	F	20's	History of visiting Daegu
Ulsan	2/22/2020	F	28	Shincheonji Church
Sejong	2/21/2020	M	32	Shincheonji Church
Gyeonggi-do	1/26/2020	M	54	Living in Wuhan, China
Gangwon-do	2/22/2020	F	30's	Shincheonji Church
Chungcheongbuk-do	2/20/2020	M	31	Contact with Shincheonji Church-related patient
Chungcheongnam-do	2/21/2020	M	25	History of visiting Daegu
Jeollabuk-do	1/31/2020	F	62	Travel history of Wuhan, China
Jeollanam-do	2/6/2020	M	46	Contact with infected patient
Gyeongsangbuk-do	2/19/2020	M	46	Contact with Shincheonji Church-related patient
Gyeongsangnam-do	2/20/2020	M	24	Shincheonji Church
Jeju-do	2/20/2020	M	22	History of visiting Daegu

Table S2. Timeline of circumstances of COVID-19 and response efforts against the spread

**Date**	**No. of cases**	**Circumstances of COVID-19**	**Response efforts against the spread of COVID-19**
20-JAN	1	The first COVID-19 case entered Korea (36-year old Chinese woman from Wuhan).	● Changes infectious disease alert form level (The national crisis management system) category fro m **level 1 (Blue) to level 2 (Yellow)**.<br>● **Quarantine and screening** measures are enhanc ed for individuals entering from **Wuhan**, China.
27-JAN	4		● Changes infectious disease alert level category fr om **level 2 (Yellow) to level 3 (Orange).**
30-JAN	5	WHO declared PHEIC.	● **Introduce the 1339 call center** as an expansion of resources available to the public.<br>● Pharmacies are given ability to check patients' tr avel histories through the patient status checking system.<br>● Recommends that anyone presenting signs of fev er or respiratory symptoms after visiting the Hub ei provinces should consult a health facility or t he call center and avoid being outdoors.
1-FEB	12		● **Supplying 720,000 masks to workplaces** that may be more vulnerable to infectious diseases d ue to large numbers of foreign employees.

2-FEB	15	Increasing number of cases among people entering South Korea from Wuhan.	● **Entry restriction for foreigners from Hubei.**<br>● Travelers from china entering Korea are directed to a separated arrival hall in Incheon international airport.<br>● Those who have been in **contact with patients** who tested positive for COVID-19 must **self-isolate for 14 days.**
3-FEB	15		● Release **"guidelines on the operation of group facilities and facilities frequented by large groups of people."**
7-FEB	24	Newly developed test kits with capability to produce results in six hours will be available at testing facilities.	● The government approved the manufacturing of first test kits for diagnosis of COVID-19.<br>● Private healthcare providers and public health institutes become designated as **additional testing facilities (46 laboratories).**<br>● The daily testing limit is increased to **3,000.**
12-FEB	28	Confirms a Patient 29, first patient without epidemiologic link.	● The government approved the manufacturing of second test kits for diagnosis of COVID-19.<br>● Release a **self-diagnosis mobile application** allowing users to monitor health conditions and access readily available information on follow-up actions.<br>● **Quarantine and screening** measures are extended to individuals from **Hong kong and Macao.**
16-FEB	29		● Increased the producing and supply of test kits for private testing institutions.<br>● The daily testing limit is increased to **10,000.**
18-FEB	31	Confirms a new COVID-19 patient at Daegu and labeled as an "super spreader".	● **Dispatch a special task force to Daegu** to "implement disease control measures with local government."
23-FEB	556	Mass transmission in the religious group at Daegu and resulted in the massive spike in COVID-19 patient numbers in the days to follow.	● Changes its infectious disease alert form level category from **level 3 (Orange) to level 4 (Red).**<br>● **Delayed new school year start by 1 week.**<br>● Begin operating the first **drive-through testing checkpoint** at Daegu.<br>● Requests **all Daegu citizens to voluntarily self-quarantine** for at least two weeks and that those displaying symptoms of the virus get tested as soon as possible.<br>● Launches the **"public reassurance hospital program,"** which separates respiratory disease patients from non-respiratory disease patients and allows patients to consult physicians and receive prescriptions virtually.
26-FEB	1,146		● Korean government secured the name list of 210,000 members of religious group.<br>● Daegu city start to **test every single member of the religious group** which has a massive infection including members who aren't exhibiting symptoms.<br>● **Increased the drive-through testing station** at more cities by the local government.
28-FEB	2,022		● Local governments begin **reporting details of epidemiological investigations and quarantine-related measures** to the KCDC.
29-FEB	2,931	Reaches the peak number of new cases per day at 909 cases.	● **Entry restriction for foreigners from 76 countries.**

			● The government guides and advise citizens to e xercise **"Social distancing"** and maintain person al hygiene until early March.
2-MAR	4,212		● **Delayed new school year start by 3 weeks.**
7-MAR	6,767		● Announce a **GPS-based APP to enforce self-qu arantine** measures, which monitor the locations of individuals who are quarantined and set off a n alarm if they leave their designated quarantine location.
9-MAR	7,382	Lack of stock of masks for the public.	● The government made a **policy to enable peopl e to purchase two masks per week per perso n**.
17-MAR	8,320		● **Delayed new school year start by 3 weeks (U ntil 6 APR).**<br>● The government declared Daegu and near city a s the first special disaster zones due to high nu mber of COVID-19 cases.
18-MAR	8,413	Increased number of influx of infected case from overseas.	● KCDC advises postponing or cancelling all on-u rgent international travel.
22-MAR	8,897		● KCDC announces all inbound travelers arriving from Europe will be tested for COVID-19 at th e airport.
25-MAR	9,137		● KCDC announces all inbound travelers arriving from United states will be tested for COVID-19 at the airport.
1-APR	9,887		● All overseas travelers arriving in Korea will required to self-quarantine for 14 days.<br>● Delayed reopening of community child care center.
9-APR	10,423		● Schools begin the academic year online.
13-APR	10,537		● KCDC announces all travelers arriving from the U.S. must be tested within three days of arrivals.
27-APR	10,738		● Individuals who fail to follow self-isolation protocols will be required to wear electronic wristbands.
1-MAY	10,774	A men positive for COVID-19, after visiting five nightclubs in Itaewon between May 1,2	● KCDC identifies **35 new cases as related to Itaewwon clubs**.<br>● Total numbers of confirmed cases related to the Itaewon cluster to 54.<br>● Seoul, Incheon, and Gyeonggi prohibit gatherings on nightlife establishments for 2 weeks.
11-MAY	10,909		● All subway passengers at Seoul are required to wear face mask during crowded hours.<br>● The ministry of education delays reopening schools by a week.
18-MAY	11,065		● All trainees who hoping to join the military are tested.
22-MAY	11,142		● All coin operated karaoke rooms at Seoul are suspended business.
25-MAY	11,206	Total confirmed case related to Itaewon nightclubs 255.	● The ministry of land, infrastructure and transport require **South Korean to wear mask on all flights and public transportation**.
29-MAY	12,757	An outbreak at the Coupang logistics centers 36 new cases at May 27	● KCDC announces the total confirmed cases related to the Coupang logistics center outbreak rise to 102.<br>● The government **holds operation of all state-run museums, galleries, and theaters** for 14 days.
4-JUN	11,629		● KCDC reports 364 new cased confirmed between May 21 and June 4 were linked to cluster infections.

10-JUN	11,902		• QR code-based registration of visitors at bars, clubs, and entertainment facilities becomes mandatory across the country.
23-JUN	12,484		• School attendance cap extended beyond June 30
10-JUL	13,338		• Government bans churches from holding small gatherings and begins QR-code entry logs for all religious facilities.<br>• All foreign arrivals from countries with high incidence of COVID-19 are required to submit certificates proving negative test results.
22-23 JUL	13,938	13 soldiers in connection with the troops stationed in Phocheon.	• Local government replace indoor cooling center for the elderly with temporary outdoor cooling areas.<br>• Two military plane were sent to bring home nearly 300 citizens from Iraq.
24 JUL	13,979	The number of new daily virus cases identified surpasses 100.	• 32 sailors on a Russia-flagged fishing vessel docked in Busan showed positive for COVID-19.<br>• Firs coronavirus case confirmed at Seoul government complex.<br>• **Korean Air resumes flights to China's Tianjin**.<br>• Fans of professional baseball and football are allowed to attend games on a limited basis.
1-AUG	14,336	KCDC report 31 daily new cases.	• Seoul grand park, reopens its indoor exhibition halls and zoo.
3-AUG	14,389		• Samsung opens a virus testing center inside its plant in Hwaseong.<br>• 4000 social academy install a QR code-based entry authentication system.
12-AUG	14,714	10 franchise employees test positive.	• **QR code-based entry log system** to be applied to wedding halls.
15-AUG	15,039	Massive anti-government rally was done with more than 10,000 participant.	• Government raises the three social distance scheme in Seoul and Gyeonggi province from level 1 to level 2, with **all indoor gatherings of 50 or more people and outdoor gatherings of 100 or more people banned effective the next day**.
18-AUG	15,761	Daily new virus cases soar by three-digit figures.	• Cumulative number of COVID-19 infection in Sarang Jeil church hits 457 cases.<br>• South Korea enhances social distancing guidelines in the metropolitan area, shutting down public and risk-prone facilities such as clubs, buffets, and sports events.
20-AUG	16,346		• Busan strengthens quarantine measures for 2nd stage of social distance.<br>• Seoul beans all assemblies of 10 or more people across the metropolitan area.
23-AUG	17,399	KCDC reports daily new virus cases spike to 397.	• Seoul city obligates face masks in all indoor areas and crowded outdoor areas.<br>• Citizens voluntarily spread **'stay home challenge'** movement to fight coronavirus.
25-AUG	17,945		• School in greater city ordered to shift online until Sep. 11.<br>• **All soldiers were ordered to wear masks at all times in public**<br>• Passengers without face masks are not allowed in taxis in Seoul.
4-SEP	20,842		• The number of new coronavirus cases stayed below 200 for the second day.<br>• Ministry of education announces that schools in

			Seoul and its surrounding cities will remain online-only until September 20.
14-SEP	22,285	KCDC reports new virus cases below 200 for a 12th straight day.	• KDCA announces only **one has coronavirus antibodies among 1,440 people in Korea**.<br>• Seoul government announces to extend a ban on rallies of 10 or more people to next month
28-SEP	23,661	50 new COVID-19 cases.	• Major state run cultural facilities reopen to the public under strict distancing and sanitary measures.<br>• Various events annually hosted by local government during Chuseok are canceled..
